# Acute Glomerulonephritis Following Systemic Scabies in Two Brothers

**DOI:** 10.3390/children11080981

**Published:** 2024-08-14

**Authors:** Flavia Chisavu, Mihai Gafencu, Ruxandra Maria Steflea, Adrian Vaduva, Floriana Izvernariu, Ramona Florina Stroescu

**Affiliations:** 1 4th Pediatric Clinic, “Louis Turcanu” Children’s Clinical and Emergency Hospital, Iosif Nemoianu 2, 300011 Timisoara, Romania; farkas.flavia@umft.ro (F.C.); steflea.ruxandra@umft.ro (R.M.S.); stroescu.ramona@umft.ro (R.F.S.); 2 Centre for Molecular Research in Nephrology and Vascular Disease, Faculty of Medicine “Victor Babes”, “Victor Babes” University of Medicine and Pharmacy, 300041 Timisoara, Romania; 3 Department XI of Pediatrics—3rd Pediatric Discipline, “Victor Babes” University of Medicine and Pharmacy, Eftimie Murgu Sq. No. 2, 300041 Timisoara, Romania; 4 Department of Microscopic Morphology-Morphopatology, ANAPATMOL Research Center, “Victor Babes” University of Medicine and Pharmacy, 300041 Timisoara, Romania; vaduva.adrian@umft.ro; 5 Department of Pathology, “Pius Brinzeu” County Clinical Emergency Hospital, 300723 Timisoara, Romania; 6 Intensive Care Unit, “Louis Turcanu” Children’s Clinical and Emergency Hospital, Iosif Nemoianu 2, 300011 Timisoara, Romania; floriananegreanu@yahoo.com; 7 Department XI of Pediatrics—1st Pediatric Discipline, Centre for Research on Growth and Developmental Disorders in Children, “Victor Babes” University of Medicine and Pharmacy, Eftimie Murgu Sq. No. 2, 300041 Timisoara, Romania

**Keywords:** APIGN, scabies, children, PRES

## Abstract

Scabies is a parasitic infestation of the skin with high prevalence in crowded spaces. In some instances, scabies becomes the underlying factor for complicated skin-borne opportunistic pathogens infections in both children and adults. Geographic area and socio-economic factors are determinants of the endemic pattern of this disease. Currently, the treatment of scabies has been under special attention. A combination of oral therapy with Ivermectin and sulfur-based ointments are the gold standard. However, caution is required in patients with kidney impairment. The renal involvement in children with scabies is mainly caused by acute glomerulonephritis. The severity of the nephritic syndrome can lead to other complications. Also, Ivermectin possesses a nephrotoxic effect. Severe hypertension can lead to neurological complications. The aim of our case report is to present two unusual complications in brothers with scabies. We report the cases of two brothers with scabies who presented with severe skin infection that developed acute post infectious glomerulonephritis (APIGN). In addition, one of the brothers presented with posterior reversible encephalopathy syndrome (PRES). The other one developed acute tubule-interstitial acute kidney injury following Ivermectin administration. The evolution of skin lesions was favorable, and kidney function returned to normal in both brothers.

## 1. Introduction

The World Health Organization (WHO) has placed scabies within the first causes of neglected tropical diseases [1]. Human scabies is a parasitic infestation with a high prevalence worldwide caused by Sarcoptes scabiei variant hominis [2]. Scabies is a skin infestation, not an infection, characterized by a very itchy rash that is often curable.

Current treatment for scabies is effective, using either topical treatments (permethrin, benzyl benzoate, and sulfur-containing compounds) and/or systemic therapies (ivermectin, moxidetin, afoxolaner) [3,4,5,6,7,8]. However, in some instances, scabies becomes the underlying factor for complicated skin-borne opportunistic pathogens infections [9,10,11,12] in both children and adults. The renal involvement is mainly caused by acute glomerulonephritis in children [1]. It seems that untreated superficial group A Streptococcus (GAS) disease is rather linked to streptococcal impetigo rather than pharyngitis in patients with scabies [13,14,15].

It is unknown why some children are more susceptible to develop acute post-streptococcal glomerulonephritis. In addition, children that are infested with scabies require longer periods of time for the infestation to be observable, as it has a heterogeneous clinical picture from erysipelas, cellulitis, impetigo, or even abscess, depending on the depth of the skin infection. One should keep in mind that scabies is extremely contagious and is associated with high recurrence rates within the same community.

The high burden of skin infections is prominent in crowded living spaces [16] and in Aboriginal children from Australia [17]. However, even though scabies is often self-limited to the skin, in some cases the severity of the complications is directly linked to the severity of the skin infection extension.

We report the cases of two brothers with scabies who presented with severe skin infection that developed acute post-infectious glomerulonephritis (APIGN). The evolution of scabies was marked by acute complications such as posterior reversible encephalopathy syndrome (PRES) in one case and acute tubule-interstitial nephritis secondary to Ivermectin treatment in the other. Resolution of the renal and neurological complications was noted following systemic and topical treatment.

## 2. Patient Information

Two brothers aged 11 and 13 years old, respectively, without remarkable past history were admitted in the Nephrology department of a tertiary Pediatric Emergency Hospital for Children from west Romania. They came from the rural area, raised by both parents, in a crowded space with eight people living in one room without current water or heat. The mother was 16 weeks pregnant at the time of the admission. All family members presented with generalized itchy skin lesions that were treated by the general practitioner with oral antihistaminic therapy and with a second generation oral cephalosporin. However, both brothers M. and A. presented with bilateral palpebral edema 5 days after the antibiotics therapy was initiated. First, they were seen in the local Emergency Department that raised the suspicion of nephrotic syndrome and redirected the cases to our hospital.

**Figure 1:**
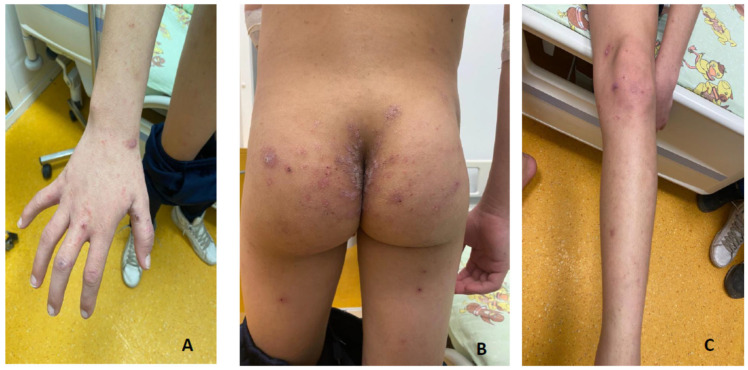
Skin lesions at admission. Legend: In this Figure above, the typical skin lesions of scabies infestation are shown. In (**A**,**C**) are the lesions extents in the upper and lower limbs. In (**B**) is shown the severity of the skin lesions in the buttocks area.

Brother M. was 13 years and 5 months old at the time of the admission. The examination revealed macular, papular, erythematous pruritic skin lesions predominantly over the upper limbs (Figure 1), bilateral palpebral edema, lower-limb edema, foamy and dark-colored urine, and high blood pressure (150/100 mmHg).

The physical examination also revealed positive water balance with approximately two kilograms over the actual weight (weight at the time of the admission was 40 kg compared to the last known weight declared by the mother of 38 kg). Laboratory investigations revealed normal hematologic parameters, normal liver function, increased serum creatinine and urea levels, 76 µmol/L and 24.16 mmol/L, respectively (reference 50–70 µmol/L and 1.4–8.3 mmol/L, respectively), with a decreased estimated glomerular filtration rate (eGFR) of 62 mL/min/1.73 m^2^ (using the revised Schwartz formula), low total serum proteins (55.7 g/L, reference 60–80 g/L), with slightly decreased albumin levels, and higher alpha1-globulins, beta-globulins, and gamma-globulins, normal C reactive protein level, but with a high erythrocyte sedimentation rate, normal lipid metabolism, and normal coagulation status. No electrolytic disturbances were recorded at admission. Also, the patient had normal immunoglobulin levels (A, G and M). The complement was consumed with low C3 and C4 levels (C3 < 0.15 g/L and C4 = 0.03 g/L, reference 0.9–1.8 g/L for C3 and 0.1–0.4 g/L for C4). The urinalysis revealed microscopic hematuria with the presence of urinary red blood cells (300 erythrocytes/μL) and proteins (0.3 g/L proteins) in the absence of a urinary tract infection. This led to a 24 h urine collection to better quantify the proteinuria that proved to be sub-nephrotic range proteinuria (38.47 mg/m^2^/h). Diuresis was preserved with a 1900 mL/24 h urine output. Initially, the patient was investigated for secondary causes of acute glomerulonephritis (AGN). Antibodies against streptolysin O were positive with a titer of 387 IU/mL (reference 0–200), which most likely reflected a streptococcal infection before admission.

Further investigations were implied in order to exclude secondary causes of AGN. Secondary causes were ruled out after a hepatic and viral serology was performed, as follows: negative antibodies against hepatitis B and C, negative human immunodeficiency virus, negative cytomegalovirus and toxoplasma, negative treponema pallidum, and also negative for COVID and Influenza virus. Autoimmune diseases were also investigated with negative antinuclear antibodies and negative rheumatoid factor. Also, anti-phospholipase A2 receptor antibodies (PLA2R) were negative. In addition, circulating immune complexes were within normal range. Quantiferon TB-gold was also negative.

The positive fluid balance was also quantified by the cardio-pulmonary radiography, with bilateral pleurisy, and also by the presence of ascites detected using abdominal ultrasound. The kidneys seemed to have normal structure, with normal echogenicity and preserved renal parenchyma.

A dermatologic consult was performed, and the diagnosis of systemic scabies was confirmed with the indication of topical treatment with sulfur-based ointments, antihistaminic medication, and isolation.

However, given the high suspicion of APIGN based on the clinical and paraclinical investigations, with nephritic syndrome, arterial hypertension, glomerular hematuria, acute kidney injury (AKI) KDIGO 2, proteinuria, and persistent low C3 and C4 levels, combined with the severity of the scabies infestation, imposed the association of systemic therapy with oral Ivermectin (6 mg per dose in two doses at 7 days apart). A kidney biopsy was performed prior to the second dose of Ivermectin that confirmed the APIGN.

The kidney biopsy was performed in the eighth day of admission under general anesthesia, with the patient in prone position. Two fragments from the right kidney cortex were obtained, one for haematoxylin-eosin (HE) staining and one for immunofluorescence staining.

Macroscopic evaluation revealed that there were two cylindrical fragments, brownish, elastic, with a 0.1 cm diameter and length of 0.3 and 1 cm, respectively.

**Figure 2:**
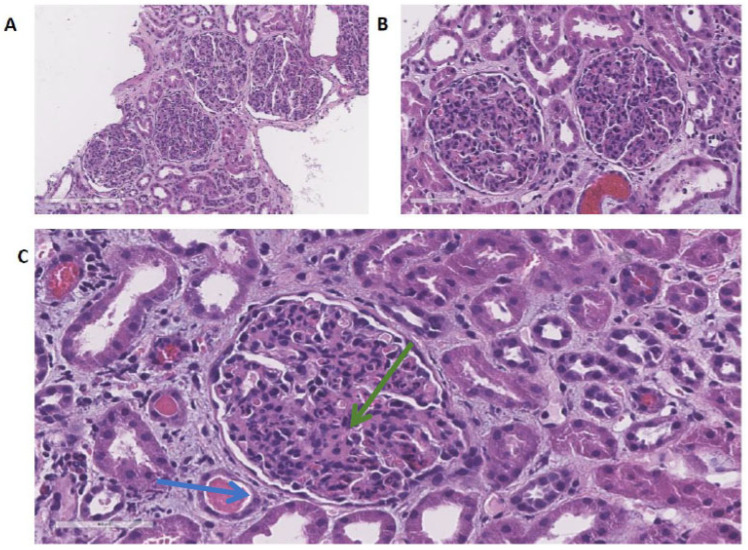
Kidney biopsy—optic microscopy with HE staining. Legend: Haematoxylin eosinophil (HE) stain, magnified by 20× (**A**,**B**) and 40×, respectively (**C**). Hypercellular glomeruli with numerous neutrophils inside the capillary loops (blue arrow), and minimal mesangial expansion (green arrow).

Microscopic evaluation of the HE staining revealed two fragments of the renal parenchyma with 32 renal corpuscles, with diffuse hyper cellularity through endocapillary and mesangial proliferation, and also with numerous inflammatory-type cells (granulocytes) infiltrating the capillary bundle—Figure 2. An increased mesangial matrix was seen along with the sporadic accentuation of the lobular design. Capillary loops were frequently obliterated. No signs of extra-capillary proliferation or necrotizing aspects. There were some hematic cylinders, a slightly periglobular edema of the interstitial space, and some inflammatory infiltrates, but with intact vessels.

**Figure 3:**
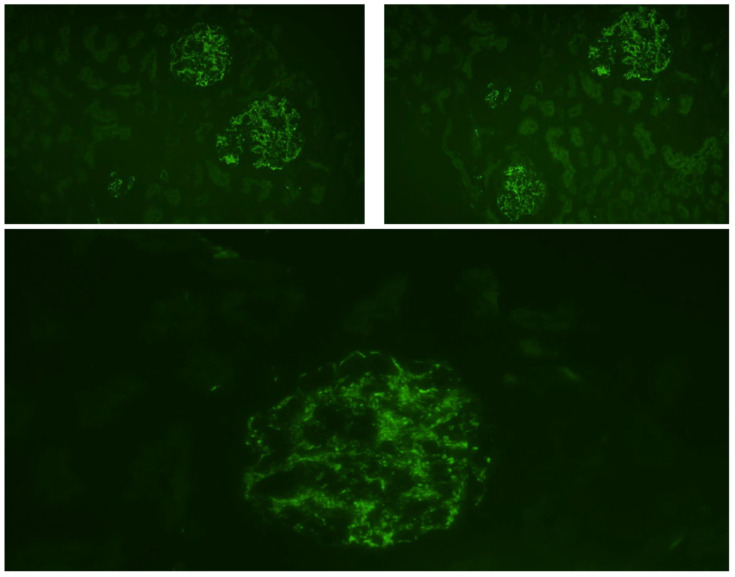
Kidney biopsy—immunofluorescence staining. Legend: Immunofluorescence staining with 20× magnification. Coarse granular deposition of C3c deposits along the glomerular basement membrane. C3c-FITC stain.

The immunofluorescence showed C3 deposits—Figure 3. The overall histopathology aspect was suggestive of an acute diffuse glomerulonephritis.

Immediately post kidney biopsy, the patient developed macroscopic hematuria secondary to a renal hematoma that persisted until discharge (7 days after kidney biopsy).

**Figure 4:**
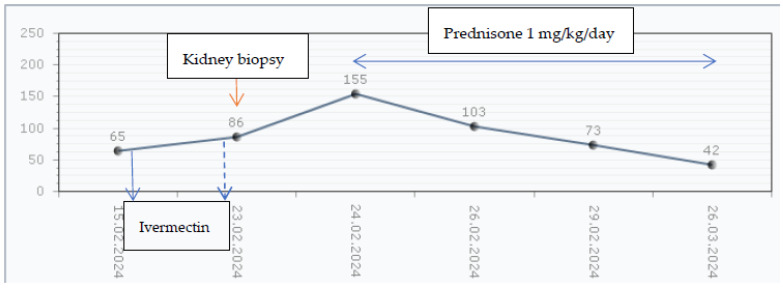
The serum creatinine levels evolution. Legend: The first dose of Ivermectin was administered in the first day of hospital stay—blue line. One should notice the descending trend in serum creatinine (eGFR = 72.55 mL/min/1.73 m^2^). The protocol in scabies requires two dose administrations. Prior to kidney biopsy, the second dose of Ivermectin was administered—dotted blue line. There was an accelerated kidney function decline in the first 48 h after administration of a nephrotoxic medication. After reaching the maximum serum creatinine level of 155 µmol/L, Prednisone therapy was initiated. The baseline serum creatinine was recorded at 6 weeks after the acute glomerulonephritis episode developed. There was a slow recovery of kidney function.

Interestingly, the patient presented worsening kidney function after the second Ivermectin dose, being framed as Ivermectin-induced nephrotoxicity, as represented in Figure 4. Thus, the tubule-interstitial AKI episode along with the persistent macroscopic hematuria and increasing proteinuria, quantified by a 24 h collection, reaching nephrotic range proteinuria (58.18 mg/m^2^/h), has led to the administration of corticosteroid treatment with oral Prednisone (1 mg/kg/day). Also, the patient continued with the calcium channel blocker (Nifedipine 1 mg/kg/day) over the corticosteroid treatment. The evolution of the skin lesions was slowly favorable after 1 month. Kidney function recovered completely, with the persistence of kidney damage markers (hematuria and low-grade proteinuria of 9.9 mg/kg/m^2^) and by achieving normal blood pressure levels for age and height without any antihypertensive treatment at the 4 weeks follow-up.

Brother A., who was 11 years and 5 months old at the time of the admission in our hospital, presented the same skin lesions as brother M., and was previously treated with antihistaminic and antibiotic therapy as well. The evolution was marked by a bilateral palpebral edema and accentuated itchy generalized rash. After the first visit in the local emergency hospital, the boys were both transferred to our hospital with the suspicion of nephrotic syndrome. The biological markers revealed AKI with serum creatinine of 75 μmol/L, urea level of 6.33 mmol/L, eGFR of 64 mL/min/1.73 m^2^, high lactate dehydrogenase level (339 U/L, reference 120–300 U/L), leukocytosis and neutrophilia, positive inflammatory markers with positive erythrocyte sedimentation rate (12 mm/h, reference < 10), and high interleukin-6 level (14.4 pg/m, reference < 7), with hematuria and traces of proteins in the dipstick urinalysis. However, during the ambulance transfer, the patient presented generalized tonic–clonic convulsions that repeated in the emergency department at arrival and also during the computer tomography (CT) scan. The CT revealed multiple hypo-dense bilateral occipital and parietal lesions in the periventricular white matter. Given the severity of the neurological manifestation, the patient was admitted in the intensive care unit (ICU).

**Figure 5:**
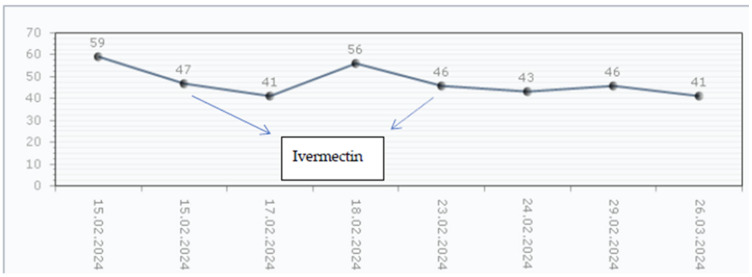
Serum creatinine dynamics in brother A. Legend: Ivermectin was administered in two doses, 1 week apart, from the first day of admission. The serum creatinine levels were on the descendent trend, with some variations during hospital stay, without, however, reaching the definition of acute kidney injury afterwards.

At admission in the ICU department, the patient was hypertensive with blood pressure of 179/118 mmHg, tachycardia (heart rate of 170 beats/minute), and decreased basal vesicular murmur bilaterally, bilateral palpebral edema, lower-limb edema, with preserved diuresis (1.44 mL/kg/h), microscopic hematuria, and multiple generalized skin lesions. Fluid overload was quantified retrospectively by weight gain (with an excess of 7 kg, from 34 kg at admission in ICU to the dry weight of 27 kg at discharge), the bilateral pleurisy seen at cardio-pulmonary radiography, and ascites detected during abdominal ultrasound. Also, the kidneys ultrasound was within normal range. Laboratory investigations revealed normal hemoglobin level, marked leukocytosis with neutrophilia (78.4% neutrophils out of 17,880/mm^3^ leucocytes), with normal liver function, decreasing until normalization of the serum creatinine and urea levels—Figure 5. Patient showed normal coagulation status, except for a high D-dimer value of 1303 ng/mL (reference < 250 ng/mL), normal lipid profile, and with metabolic acidosis, high lactate (4.1 mmol/L, reference < 1.2 mmol/L), hypocalcaemia, and normal serum proteins (64.6 g/dL, reference 60–80).

C3 levels were low, as in the case of his brother. A 24 h urine collection revealed a nephritic range proteinuria (27.24 mg/m^2^/h). Antibodies against streptolysine-O were also positive (438 U/L). There were no pathological modifications of the immunoglobulin A, M, or G. Also, hepatic serology was negative for hepatitis B and C. The HIV test was negative. Cytomegalovirus, toxoplasma, treponema, and EBV serology were also negative. COVID-19 and Influenza infections were also excluded. The negative Quantiferon excluded tuberculosis. Autoimmune diseases were also excluded: negative antinuclear antibodies and negative rheumatoid factor. Also, the antibodies against anti-phospholipase A2 receptor were negative, as well as the immune complexes.

**Figure 6:**
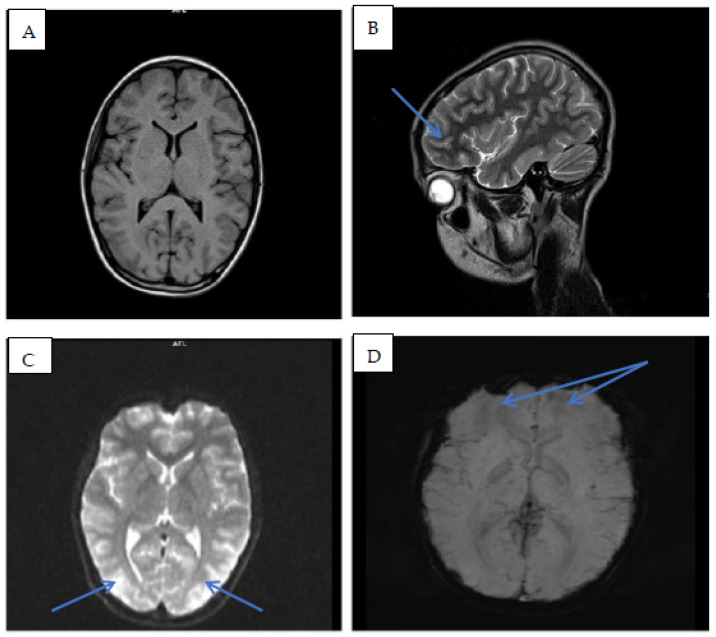
Magnetic resonance imaging with contrast. Legend: Brain MRI performed on the second day after onset of symptoms detected increased signal on Flair- and T2-weighted imaging in the subcortical and cortical regions of the parietal-occipital areas (axial FLAIR images (**A**,**C**,**D**)). Also, the superior frontal area showed the same changes (sagittal view (**B**)), pointed out by the blue arrows. However, normal diffusion without hemorrhages were identified (**A**). The diagnosis of PRES was made after imaging was performed.

Antibiotic treatment was initiated in the ICU settings for the extent of the infected skin lesions with a broad-spectrum antibiotic (Meropenem for 10 days). Also, due to the severity of the neurologic symptoms, the patient was initially treated with intravenous Midazolam to control their seizures, and afterwards continued with oral Levetiracetam and intravenous corticosteroids with Dexamethasone. The severe hypertension was controlled with a calcium channel blocker. The electroencephalogram confirmed the comitial crises with medium-voltage theta rhythm with isolated delta waves discharged during examination. A magnetic resonance imaging (MRI) with contrast was performed on the second day of hospitalization that showed the changes in T2 hyper signal, with cortical-subcortical changes in the superior frontal area and bilateral in the parietal–occipital area, that was relatively symmetrical, being framed as posterior reversible encephalopathy syndrome—Figure 6. He also received oral Ivermectin (6 mg/dose) in two doses one week apart, along with sulfur and 25% benzyl-benzoate-based ointments, as did his brother (see Figure 5).

However, in the face of nephritic syndrome, microscopic hematuria, severe hypertension, and consumed complement, doubled by the severity of the neurological manifestation, in a patient with scabies raised the suspicion of an APIGN. This is why the patient underwent general anesthesia on the eighth day of hospital stay when the kidney biopsy was performed. However, the results could not confirm the lesions seen in brother M., as the biopsy did not comprise any renal parenchyma.

The evolution was marked by the resolution of the neurological manifestation once the blood pressure was controlled, with a slow but favorable resolution of the skin lesions and the resolution of kidney damage markers at 4 weeks follow-up (hematuria, proteinuria).

## 3. Follow-Up and Outcomes

Both brothers were followed-up at four weeks. The evolution of the biological parameters were obtained and detailed in Table 1. The C3 and C4 values returned to normal at four weeks after the onset of the symptoms. Normalization of blood pressure occurred without further antihypertensive treatment, and normal kidney function occurred alongside the partial resolution of the kidney damage markers (proteinuria, albuminuria, and hematuria).

## 4. Discussion

This is the first case report of familial aggregation of AGN in two brothers with systemic scabies. Although scabies is highly contagious among family members, herby we presented the renal and neurological involvement secondary to infected skin lesions caused by scabies. Also, we report the potential drug-induced toxicity of Ivermectin in patients with scabies. One of the major limitations is the lack of kidney tissue in the patient with PRES for histological quantification of the kidney alterations and the lack of follow-up imaging after the resolution of the skin lesions.

Currently, the treatment with oral Ivermectin and sulfur-based ointment are considered the gold standard for scabies infestation [3,4,5,6,7,8]. Alternatively to oral treatment with Ivermectin, ointment treatment with Permethrin has equivalent efficacy. However, Permethrin was not available at the time of admission [18]. Even though Permethrin seems to be superior to Ivermectin, the adherence is higher for the latter, as it is cheaper and more accessible [18]. Ivermectin proves to be efficient; however, one should keep in mind the possible nephrotoxic effect that can occur and the age limitation [19,20]. Although there are no studies in children, it seems that Ivermectin-induced AKI is more probable after the second dose, as we described earlier. However, we cannot support this based on a single case report, especially in a child who already had decreased kidney function. In addition, Ivermectin achieves higher concentrations in children as they present a relatively higher distribution volume as opposed to adults, mostly when treatment is administered per kilogram weight (200 µg/kg) [21].

Left untreated, the skin lesions in scabies can become infected. The main etiologies are the spectrum of group A Streptococcus (GAS) that seems to be responsible for the APIGN. Most commonly, the downstream complications of scabies are caused by *Staphylococcus aureus* and *Streptococcus pyogenes* [12]. In addition to the high prevalence of APIGN reported in the aboriginal children from Australia [2], there are few studies that report AGN in children. In addition to treating the underlying disease, AGN treatment is supportive. In our cases, the APIGN was presumed to be secondary to GAS infection as we excluded other secondary causes of AGN. Even though the ASLO levels were slightly elevated, without positive throat and skin lesion cultures, we considered the etiology to be most likely a GAS infection, especially as we could not compare the current ASLO levels to a previous one. Also, both brothers received antibiotic treatment prior to hospitalization and during admission.

The long-term consequences are linked to persistent proteinuria and a high risk of progressing to chronic kidney disease later in life [22]. In addition, kidney biopsy can be useful in clinical practice depending on the histological pattern identified, as has been previously described [23]. Given the fact that the kidney biopsy was performed in acute settings, with the endocapillar mesangial pattern identified using an optic microscopy, the renal prognosis seems to be overall associated with renal recovery [23]. However, it has been described that the endocapillar mesangial patter evolves towards the mesangial pattern, which in turn is recognized by C3 staining alone in immunofluorescence [23].

Neurologic impairment has been described in patients with scabies, with a variety of symptoms from convulsions to stroke [24,25]. PRES associated with scabies in children has never been reported. Furthermore, PRES is an underdiagnosed illness, perhaps due to the limited accessibility of superior imaging, especially in the acute settings. The multifactorial etiology of PRES has been explored in the existing literature [1,2,3,4,5,6,7,8,9,10,11,12,13,14,15,16,17,18,19,20,21,22,23,24,25,26,27,28,29,30]. Per Laetitia della Faille et al. found kidney failure and chemotherapy as predictors of PRES [30], besides hypertension, autoimmune diseases, or sepsis [26,27,28,29]. However, not all patients with hypertension will develop PRES [31]. Thus, maybe the lack of reported PRES in children with APIGN secondary to scabies infestation is underreported. In addition to the severity of the neurological manifestations in PRES, the prompt control of blood pressure often leads to the resolution of the neurological impairment. Although there is no consensus regarding antiepileptic treatment for seizures in children with PRES, during the acute phase of PRES, it seems reasonable to intervene [32,33]. However, once the PRES resolves, therapy can be discontinued in the absence of proven epilepsy [29]. Early imaging and treatment of PRES can improve the long-term outcomes. In patients with scabies and nephritic syndrome, and those with severe hypertension, the physicians should be aware of PRES.

The severity of the skin lesions in patients with scabies infestation vary among individuals. These lesions increase the risk of GAS. Further, GAS infection could lead to APIGN with different degrees of renal involvement. PRES is a severe complication secondary to sudden and severe arterial hypertension. It seems that there is a link between the severity of the renal involvement and possible complications. Within this case report, we described renal involvement secondary to APIGN and also secondary to the treatment of scabies.

In conclusion, with this case report, we would like to draw attention to the possible complications of systemic scabies in children and the complications associated with the current treatment with Ivermectin. The clinicians must be aware of the potential nephrotoxic effect of Ivermectin in children with kidney impairments. Prompt recognition of organ dysfunction is required in order to improve the short- and long-term outcomes. Although PRES is reversible when the underlying disease is framed and treated, it possesses high morbidity in pediatric settings.

## Figures and Tables

**Table 1:** Biological parameters at the time of the admission and at follow-up.

	Brother A.		Brother M.		Reference
	1 Day of Admission	4 Weeks Follow-Up	1 Day of Admission	4 Weeks Follow-Up	
ASLO	387	279	438	411	0–200 U/L
C3	<0.15	1.10	<0.15	1.11	0.9–1.8 g/L
C4	0.22	0.39	0.03	0.4	0.1–0.4 g/L
Creatinine	59	41	65	42	46–70 µmol/L
eGFR	82	118	72.55	120	>90 mL/min
Hematuria	+++	negative	+++	+++	No traces
Urinary sodium	164	-	110	-	30–300 mmol/L
Proteinuria	752	128	1182	290	<150 mg
Urea	7.2	4.04	21.19	4.16	1.4–8.3 mmol/L

Legend: The biological parameters that require follow-up in case of acute glomerulonephritis. ASLO = anti streptolysin O antibodies; U = units; L = liter; g = grams; µmol = micromoles; mg = milligrams; +++ represents 300 erythrocytes per filed; eGFR = estimated glomerular filtration rate measured in milliliters per minute per 1.73 m^2^.

## Data Availability

All of the data regarding these cases are present in the article.
